# Modeling of the brain-lung axis using organoids in traumatic brain injury: an updated review

**DOI:** 10.1186/s13578-024-01252-2

**Published:** 2024-06-22

**Authors:** Jong-Tae Kim, Kang Song, Sung Woo Han, Dong Hyuk Youn, Harry Jung, Keun-Suh Kim, Hyo-Jung Lee, Ji Young Hong, Yong-Jun Cho, Sung-Min Kang, Jin Pyeong Jeon

**Affiliations:** 1 Institute of New Frontier Research, Hallym University College of Medicine, Chuncheon, 24252 Republic of Korea; 2 Department of Green Chemical Engineering, Sangmyung University, Cheonan, 31066 Republic of Korea; 3 Department of Periodontology, Section of Dentistry, Seoul National University Bundang Hospital, Seongnam, 13620 Republic of Korea; 4 Division of Pulmonary and Critical Care Medicine, Department of Medicine, Hallym University College of Medicine, Chuncheon, 24252 Republic of Korea; 5 Department of Neurosurgery, Hallym University College of Medicine, Chuncheon, 24252 Republic of Korea

**Keywords:** Traumatic brain injury, Brain-lung axis, Brain organoids, Lung organoids, Organ-on-a-chip

## Abstract

Clinical outcome after traumatic brain injury (TBI) is closely associated conditions of other organs, especially lungs as well as degree of brain injury. Even if there is no direct lung damage, severe brain injury can enhance sympathetic tones on blood vessels and vascular resistance, resulting in neurogenic pulmonary edema. Conversely, lung damage can worsen brain damage by dysregulating immunity. These findings suggest the importance of brain-lung axis interactions in TBI. However, little research has been conducted on the topic. An advanced disease model using stem cell technology may be an alternative for investigating the brain and lungs simultaneously but separately, as they can be potential candidates for improving the clinical outcomes of TBI.

In this review, we describe the importance of brain-lung axis interactions in TBI by focusing on the concepts and reproducibility of brain and lung organoids in vitro. We also summarize recent research using pluripotent stem cell-derived brain organoids and their preclinical applications in various brain disease conditions and explore how they mimic the brain-lung axis. Reviewing the current status and discussing the limitations and potential perspectives in organoid research may offer a better understanding of pathophysiological interactions between the brain and lung after TBI.

## Background

Traumatic brain injury (TBI) is a major cause of mortality and morbidity worldwide. Although precise data on the worldwide incidence of TBI are not yet available, 69 million people are estimated to experience TBI per year [1]. In the United States, about 1.5 million subjects experience TBI, and 50,000 of them die [2]. Analyses of Korean National Health Insurance data between 2008 and 2017 showed that the age-adjusted TBI incidence per 100,000 people increased until 2010 but showed a tendency to decrease thereafter (571.3 cases in 2008, 638.1 cases in 2010, and 475.8 cases in 2017) [3]. However, the incidence of TBI is still increasing in older patients over the age of 70, and their high mortality rate is becoming a clinical problem [3]. Elderly TBI patients were reported to exhibit more severe deterioration and less improvement in functional outcomes over the first 5 years after the event [4]. In addition, even with mild TBI severity, elderly patients are more likely to have physical symptoms than younger patients [5]. In actual clinical practice, elderly TBI patients often have underlying co-morbid diseases. Thus, even with isolated TBI, their prognosis is likely to be poorer compared to young TBI patients. Mortality after TBI is closely related to brain damage and to the conditions of other organs, especially the lungs. Even without direct pulmonary injury due to trauma, pulmonary complications, such as pneumonia, pleural effusion, acute lung injury, acute respiratory distress syndrome (ALI/ARDS), and neurogenic pulmonary edema, are observed [6]. TBI patients with pneumonia and acute respiratory failure demonstrated a higher mortality risk of 1.73-fold, even after adjusting for clinical and demographical factors [7]. These results suggest that treatments for patients with TBI should be performed considering the interactions between the brain and lungs, which is also called the brain-lung axis.

**Fig. 1:**
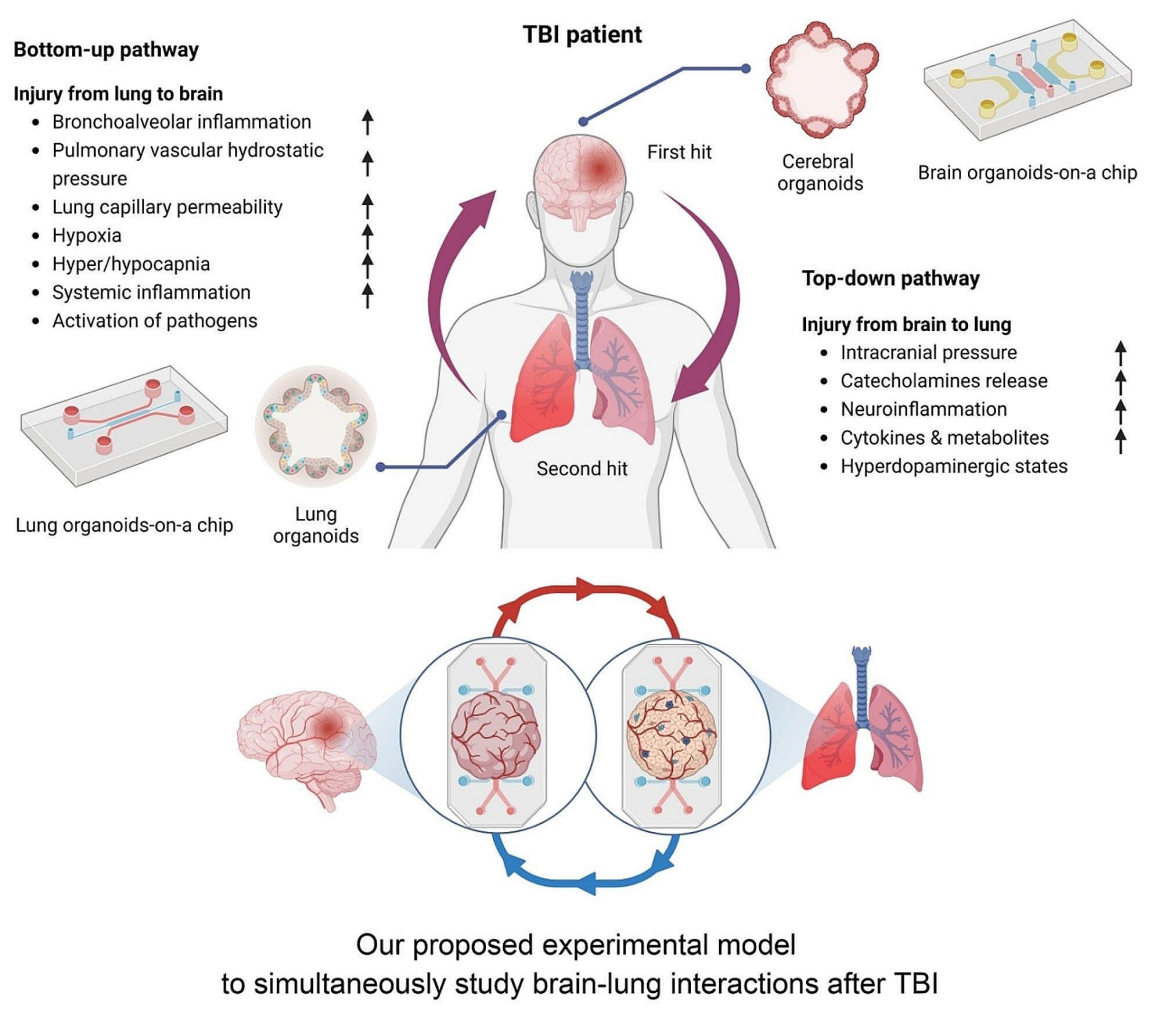
Schematic representation of brain-lung interactions after traumatic brain injury (TBI) and the concept of different organ models to study simultaneous interactions including bottom-up and top-down pathways of brain and lung axis

Rapid sympathetic increases following TBI can trigger acute pulmonary edema. More specifically, sudden increased intracranial pressure (IICP) leads to upregulated sympathetic tones on blood vessels and systemic vascular resistance, resulting in left ventricular failure and subsequent neurogenic pulmonary edema [8]. Pulmonary dysfunction can also aggravate cerebral hypoxia, resulting in a vicious cycle that further exacerbates IICP [9]. Pulmonary responses can occur following not only moderate-to-severe TBI but also mild TBI. Lim et al. reported that mild TBI showed cellular infiltration of the alveolar space with morphological changes and interstitial edema [10]. Increases in tumor necrosis factor (TNF)-α levels were evident in the lungs 6 h after TBI [10]. These findings suggest that TBI can contribute to acute alveolar structural changes with interstitial inflammation, worsening the prognosis of patients with TBI. Besides the acute phase, the brain and lungs interact in immune processes. Before autoreactive T cells enter the central nervous system (CNS) via systemic circulation, they temporarily stay in the lungs and mature in local lymph nodes [11, 12]. Hosang et al. reported that a dysregulation of the lung microbiome was associated with CNS-specific autoimmune disorders [13]. In their study, the administration of neomycin caused a shift in microbiota with lipopolysaccharide-enriched phyla, consecutively upregulating type II interferons and immune cell recruitment in the brain [13]. These findings suggest that the brain and lung cannot be considered separately after TBI. Thus, the development of diagnosis and treatment should be approached by considering both the brain and lung, as well as their interactions. Due to the advance in stem cell research, organoids, which are three dimensional (3D)-like structures, have been increasingly used to study various diseases, treatment methods, and disease pathways in vitro, including TBI [14–16]. Advanced stem cell technologies also enable studies on interactions between the brain, lungs, gut, and other organs [17]. Over the past five years, we have performed research on various types of organoids and organ-on-a-chip platforms [16, 18–20]. Based on our experience with organoids, we provide an updated review of research examining brain and lung organoids and discuss their interactions after TBI to improve the understanding of clinicians and relevant researchers (Fig. 1). Their clinical application and future perspectives are also discussed.

### Cerebral organoid generation

Significant progress has been made in cerebral organoid (CO) generation from the 3D aggregates of embryonic stem cells (ESCs) as pluripotent stem cells (PSCs). COs derived from PSCs generated by protocols inspired by human brain development can recapitulate brain development and function in in vitro conditions at the early stage to highly control neurodevelopment [21]. Although each method describing neural differentiation into major cell types within the brain was established in in vitro two-dimensional (2D) conditions with induction cues [22–24], once the cells differentiated into neural subtypes from PSCs, neural stem cells (NSCs) or neural progenitor cells (NPCs) could not obtain 3D architecture or the complexity of the neural cell subtypes of the desired brain-like organ [25]. Therefore, most scientists currently define 3D COs as reflecting diverse cell complexity, architecture, and physiological function by mimicking human organ models or disease models [24, 26, 27].

**Fig. 2:**
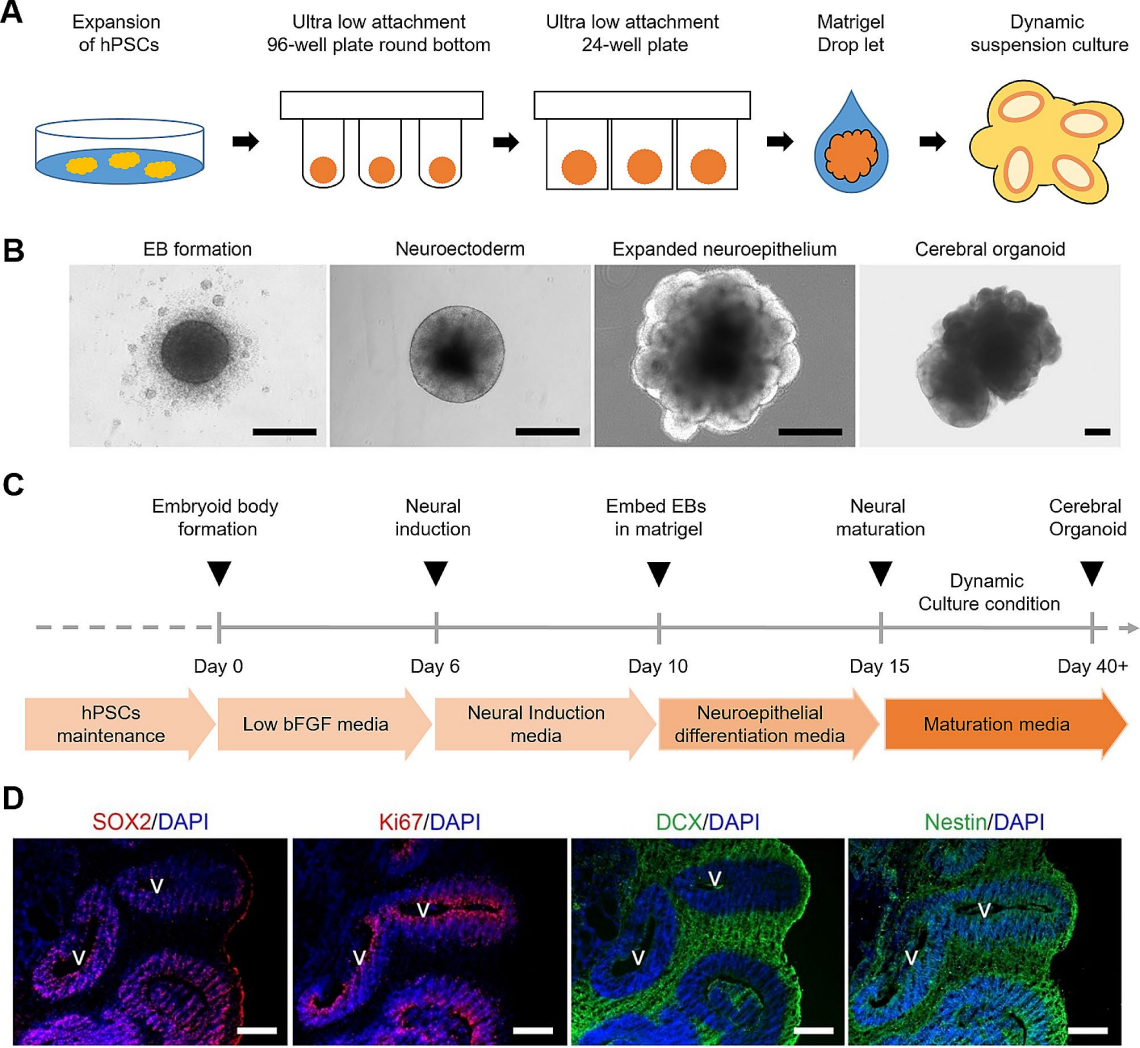
Cerebral organoid culture concept using human pluripotent stem cells. **(A)** Schematic illustration of 3D dynamic culture platforms for cerebral organoids (COs). hPSCs, human pluripotent stem cells. **(B)** Representative images showing various morphological changes at each stage. Scale bars are 300 μm. EB, embryonic bodies. Reproduced with permission [16]. Copyright 2022, Elsevier. **(C)** Schematic timeline of generating COs. bFGF, basic fibroblast growth factor. **(D)** Immunofluorescence staining for SOX2, Ki67, DCX and nestin in COs. Scale bars are 200 μm. All cell nuclei were stained with DAPI. SOX2, SRY-Box transcription factor; Ki67, proliferation marker; Doublecortin (DCX), neuronal precursor cell marker; nestin, neural stem cell marker; V, ventricle-like structures. DAPI, 4′,6-diamidino-2-phenylindole. Reproduced with permission [16]. Copyright 2022, Elsevier

As described in the section of introduction, we focused on recent insight to understand fundamental CO culture methods and their potential use in TBI, as well as limitations to overcome. The general process of generating COs is described in Fig. 2A. In the case of whole brain organoids as COs, the protocols and culture conditions proposed by Lancaster et al., which are a rapid development method using a spinning bioreactor or orbital shaker, are widely used in human neurobiology research [27]. Human brain development is an extraordinarily complex process orchestrated by the fine adjustment of autocrine and paracrine growth factors, extracellular matrix (ECM), and cell-to-cell interactions [28]. Thus, the first step in generating COs is to form an embryonic body (EB) that is beginning to differentiate to neuralization from PSCs by double inhibition of the Smad pathway [29]. About 9000 human PSCs (hPSCs) were cultured with serum-free medium containing low basic fibroblast grwoth factor for floating EB aggregates using ultra-low attachment 96-well round-bottom plates. In this step, some signaling factors or inhibitors can be added into the media to avoid undesirable lineage differentiation, such as mesendoderm, non-neural ectoderm, and mesodermal lineages [30–32]. The regulation of signaling molecules for the fate of cells in the development of the CNS and specific brain regions is a critical step. The second step was neural induction to promote neurogenesis of the EB until a size of about 500 μm in diameter was obtained. In this stage, EB cells can initiate neurogenesis and differentiate into neural progenitors that cells secrete growth factors or morphogens through self-organization [33]. Borello et al. reported that these coordinated morphogens and gradients determined the anteroposterior and dorsoventral axes through various secreted molecules, including fibroblast growth factor, wingless-type MMTV integration site family, sonic hedgehog, transforming growth factor-β/bone morphogenetic proteins, and retinoic acid signaling pathway [34]. The third step was promoting neuroepithelial (NE) cells, which facilitate the proliferation, migration, and differentiation of neurogenic progenitor cells by providing additional ECMs. In an environment where collagen, laminin, and Matrigel are provided as ECMs, EB develops more into cortical NE tissue and neural tube-like formations. The last step was full maturation into cortical neurons. However, long-term cultivation for the mature COs is difficult to make larger due to necrosis of internal cells, even if dynamic cultivation is performed by a spinning bioreactor or orbital shake [35]. Previously, we reported our experience with CO generation using a modified procedure and confirmation of the histologic characteristics of the structure and cell diversity, as presented in Fig. 2 [16]. We could find ventricle-like structures in the COs, which is the neural stem cell niche that promotes neurogenesis in the brain. The NSCs in the niche expressed early development stage markers that included nestin, SOX2, and PAX6, and the cells showed high proliferative activity.

Since neurogenesis was first discovered to occur in certain regions of the adult mammalian brain and the isolation of NSCs [36–38], many attempts have been made to treat neurological diseases clinically. Technical difficulties and ethical problems aside, the biggest drawback to research is the lack of an animal model that reflects the human cerebral cortex. In this respect, CO research is expected to be a new alternative method to overcome such limitations. Currently, COs are widely used to identify pathological mechanisms in brain microcephaly [27, 39, 40], the influence of Zika virus infection [41–43] schizophrenia [44], autism spectrum disorder [45], and Parkinson’s disease [46, 47]. They also show potential as a therapeutic agent for the reconstruction of the cerebral cortex following TBI because COs are roughly similar to fetal brains at the development stage. A summary of the various attempts to restore neuronal cells and physiological functions by transplanting COs into the brain cortex is presented in Table 1. In the future, we think that COs will be actively studied, focusing on interactions with other organs, including the lung, instead of the brain alone.

**Table 1:** Preclinical trials of the transplantation of cerebral organoids (COs) into the brain cortex

Cell source	Transplanted time	Brain region-specific markers	Disease model	Delivery lesion	Evaluation date	In vivo differentiation	Result	Ref.
hESCs	50 days	PAX6+, nestin+, TBR2+, SOX2+, PKC-λ+, DCX+, HOPX+, NCAM+, Ki67+, NeuN+, FOXG1+, TUJ1+, TBR1+, BRN2+, CTIP2+, SATB2+, FOXP2+, GLU+, MAP2+, GABA+	Photothrombotic stroke model using rose Bengal solution in NOD-SCID mice	Microinjection into the forelimb motor cortex (PT-1) or parietal cortex (PT-2)	45–180 days	PAX6+, SOX2 + progenitor cells at 45 days; TBR1+, FOXP2+, CTIP2 + deeper-layer neurons, SATB2+, BRN2 + and NeuN + upper layer neurons, CaMKII + pyramidal neurons at 60 days; SYN + synaptic neurons at 80 days	hCOs survived, differentiated into target neurons, repaired infarcted tissue, sent axons to distant brain targets, and integrated into the host neural circuit; restored sensorimotor function	[117]
hESCs	80–88 days	PAX6+, MAP2+, CTIP2+, SATB2+	Aspiration cavity in Long Evans rats with cyclosporine A	Border of primary and secondary visual cortexes	1, 2, and 3 months	PAX6+, NeuN+, FOXG1+, TBR1+, CTIP2+, CUX1+, SATB2+, GFAP + and Olig2+	A cellular complement of hCOs supported neuronal function; hCOs were functionally integrated into host brain circuits and a visual system	[118]
hiPSCs	30–60 days	PAX6+, FOXG1+, NeuN+, TUJ1+, TBR1+, TBR2+, SATB2+, CTIP2+, BRN2+	Stereotactically transplanted in early postnatal athymic rats	Primary somatosensory cortex (S1)	up to 12 months	PPP1R17 + cortical progenitors, NeuN + neurons, SOX9 + and GFAP + glial lineage cells or PDGFRα + oligodendrocyte progenitor cells, SATB2+, CTIP2 + cortical layer subtypes	hCOs developed into mature cell types in vivo; hCOs integrated both anatomically and functionally into rodent neural circuits; hCOs modulated the activity of rat neurons to drive behavior	[119]
hESCs	56 days	42 days: SOX2+, Ki67+, DCX+, nestin+, FOXG1+, PAX6+; 98 days: CTIP2+, NeuN+	TBI model in mice with cyclosporine A	Retrosplenial cortical region	7–14 days	TUJ1 + neuron, and CTIP2+, NeuN + cortical neuron	hCOs survived and engrafted in the cortex; the survived cells were differentiated into immature neurons, but not astrocytes or oligodendrocytes; improved cognitive function	[16]
hiPSCs	40 days	SOX2+, TUJ1+, Ki67+, TUJ1+, FOXG1+, TBR1+; GFAP+, MAP2 + at 220 days	CCI model to induce TBI in hCOs	N/A	7 days	Upregulation of astrogliosis, neuronal loss, and metabolic changes	Recommended to perform CCI in hCOs not earlier than 150 days in vitro if the investigator wants to study the consequences of CCI in mature post-mitotic neurons and astrocytes.	[120]
hESCs	58 days	DCX+, TUJ1+, SOX2+, Ki67+, PAX6+, TBR1+, CTIP2+, FOXP2+, SATB2+, BRN2+	TBI model in SCID mice	Left parietal cortex	2 months	TBR1+, TBR2+, FOXP2+, and CTIP2 + cortical cells; HOPX + neural progenitor cells; BRN2+, STAB2 + neurons	Brain lesion area filling; detectable electrophysiological activity of implanted cells; reduced glial scarring; hCOs matured and differentiated into neuronal cells; improved memory and learning ability	[121]
hiPSCs	44 days	SOX2+, TUJ1+, FOXG1+, TBR1+, SATB2+, MAP2+, GFAP+	CCI model to induce TBI in hCOs	N/A	7 days	Decreased MAP2 + neurons and increased GFAP + astrocytes; increased nestin + cells	Recapitulated the primary pathological changes in TBI; novel model approach for TBI in vitro	[14]
hESCs hiPSCs	40 days	FOXG1+, FOXP2+, CTIP2+, TBR1+, HOPX+, Ki67+, SOX2+, nestin+, DCX+, PAX6+, GAD67+, GLU+	Injection into SCID mice using a pulled glass pipette	Medial prefrontal cortex	1–5 months	SOX2+, FOXG1+, NCAM+, NeuN and TUJ1 + at 1 month; TBR1+, FOXP2+, SATB2+, BRN2+, GLU+, VGLUT1 + and SYN + at 3 months	hCOs extended long projections into distal brain regions and acquired electrophysiological maturity; functionally integrated into mouse neural circuits	[122]
hESCs	55 days	TUJ1+, SOX2+, FOXG1 + TTR+, nestin+	MCAO model in SD rats with cyclosporine A	Motor cortex	4 weeks	nestin+, TUJ1+, GFAP+, SATB2+, Olig2+, HB9+	Significantly reduced infarct volume; motor cortex region-specific reconstruction; improved neurological motor function	[123]
hESCs	42–70 days	FOXG1+, PAX6+, CTIP2+, Ki67+, MAP2+, SATB2+	Aspiration cavities in SCID mice or cynomolgus monkeys	Frontal and parietal cortices	12 weeks	PAX6+, Ki67+, CTIP2+, SATB2+, TUJ1+	hCOs extended axons along the host corticospinal tract; 42-day hCOs extended more axons than 70 days hCOs but caused graft overgrowth; axons of 70-day hCOs could be extended by delayed transplantation into the brains of nonhuman primates	[124]
hESCs	55 or 58 days	55 days: SOX2+, nestin+, TUJ1+, FOXG1+, TTR+; 85 days: nestin+, TUJ1+, GFAP+, NeuN+, FOXG1+, TTR+	TBI model in SD rats with cyclosporine A	Motor cortex	8 weeks	nestin+, TUJ1+, GFAP+, TBR1+, SATB2+, Olig2+, ChAT+, vGlut1+	55-day hCOs were better transplantation donors for brain injury, which Increased neurogenesis and cell survival better than 85-day hCOs; improved motor function and reduced brain injury	[125]
hESCs	42 days	Ki67+, SOX2+, CTIP2+, DCX+	Cortical lesion by removing 1 mm^3^ piece in CD1 mice	Frontoparietal cortex	2 and 4 weeks	DCX + neuroblasts, TBR2 + intermediate progenitor cells, CTIP2 + neurons, neurofilament heavy chain + neurons	Increased cell survival, robust vascularization, and neuronal differentiation	[126]
hESCs	40–50 days	PAX6+, CTIP2+	Aspiration cavity in NOD/SCID mice	Retrosplenial cortex region	0.5-8 months	SOX2 + NPC cells, NeuN+, SMI312 + neuronal cells at 50 day; SYN+, PSD95 + synaptic neuron at 50 days; Olig2 + oligodendrocytes, GFAP + astrocytes; but not IBA + microglia	Increased neuronal differentiation and maturation; developed functional synaptic connectivity and neuronal activity between grafted and host brain	[127]

*hESCs, human embryonic stem cells; hiPSCs, human induced pluripotent stem cells; TBI, traumatic brain injury; MCAO, middle cerebral artery occlusion; CCI, controlled cortical impact; NOD/SCID, nonobese diabetic/severe combined immunodeficiency; SCID, severe combined immunodeficiency

**SOX2 indicates proliferated neural progenitor cells marker; PAX6, dorsal telencephalic progenitor marker; KI67, proliferation marker; FOXG1, telencephalic marker; FOXP2, developing neuronal subset cell marker; CTIP2, subcerebral projection neuron marker; SATB2, callosal projection neuron marker; DCX, immature neuronal marker; BRN2, neuronal subtype progression marker in the cortex; CUX1, cortical layer marker; PPP1R17, cortical progenitor marker; TBR1, preplate marker; TBR2, intermediate progenitor marker; TUJ1, immature neuron marker; MAP2, neuronal marker; NeuN, mature neuron marker; GFAP, astrocyte marker; Olig2, oligodendrocyte lineage-specific marker; PDGFRα, oligodendrocyte progenitor cells; PKC-λ, apical marker; HOPX, outer radial glial cells marker; NCAM, neural cell adhesion molecule marker; GABA, GABAergic neuronal marker; GAD67, GABAergic neuron marker; CaMKII, pyramidal neuron marker; HB9, motor neuron marker; SYN, synaptic vesicle marker; PSD95, synapse-associated protein marker; SMI312, neurofilament marker; ChAT, cholinergic neuronsmarker; vGlut1, glutamatergic neuron marker; GLU, glutaminergic neuron marker; IBA, microglia marker

### Lung organoid generation

**Fig. 3:**
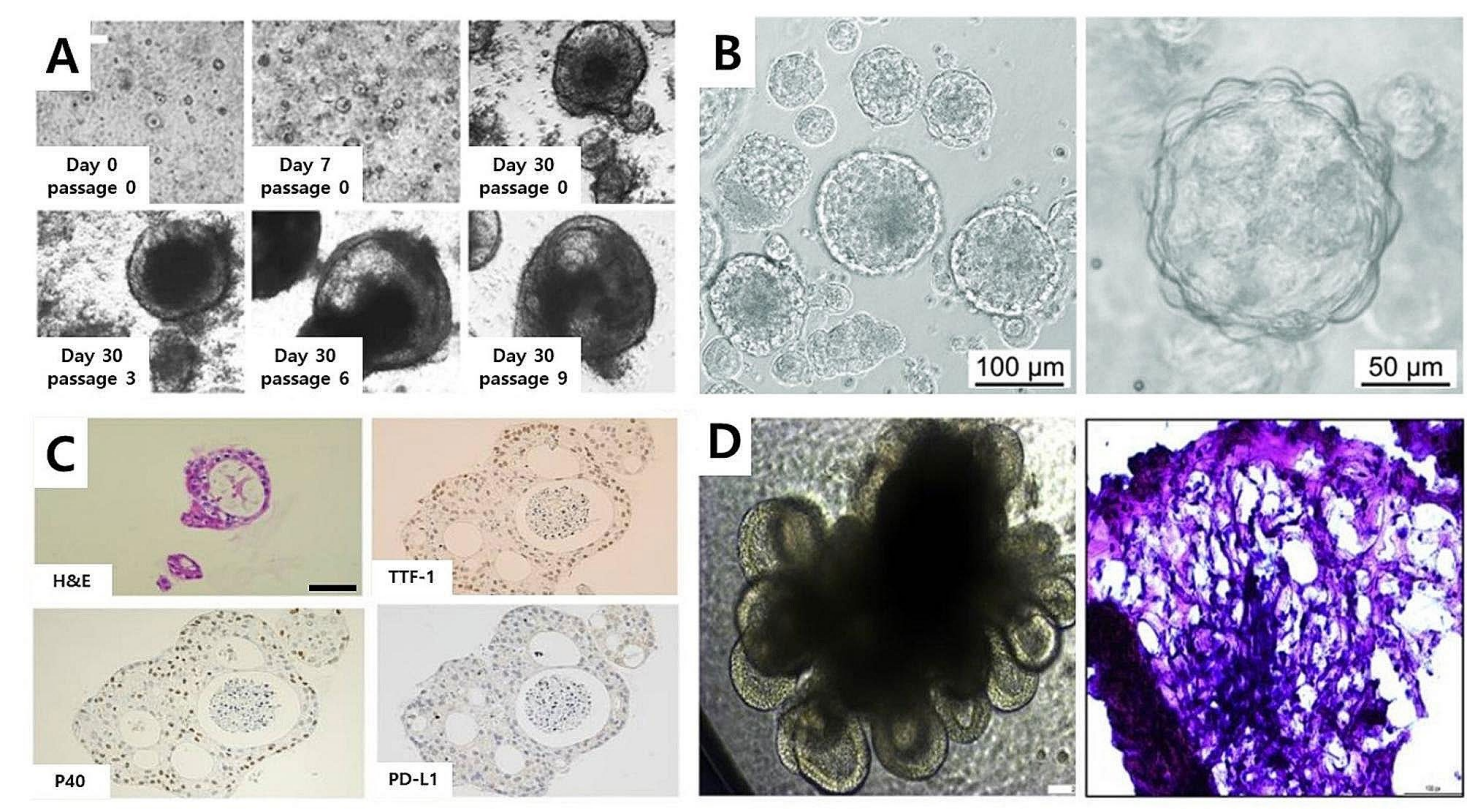
Culture of lung organoids using various cell lines and methods. **A**. Lung cancer organoids (LCOs) generated using Matrigel and minimum basal medium for the lung cancer biobank. Representative images of LCOs cultured long-term. Reproduced with permission [52]. Copyright 2019, Nature Publishing Group. **(B)** Bright-field images of lung organoids derived from the HTII-280 + fraction cultured in a complete alveolar medium. Reproduced with permission [61]. Copyright 2022, American Physiological Society. **(C)** Lung organoids from cryobiopsy specimens. Hematoxylin and eosin (H&E) staining and immunohistochemical (IHC) staining of LCOs. IHC staining profiles, including TTF-1, p40, and PD-L1. Reproduced with permission [62]. Copyright 2023, MDPI. **(D)** Human induced pluripotent stem cell (iPSC) differentiation into 3D lung organoids for modeling SARS-CoV-2 infection. Representative phase-contrast image of lung organoids at 60 days. H&E staining of 60-day lung organoids showing alveolar-like morphology. Scale bars represent 200 μm (left) and 100 μm (right). Reproduced with permission [65]. Copyright 2021, Elsevier

Since human lungs are different from animal lungs, existing animal models do not reflect human conditions. Thus, supplemental in vitro experiments should inevitably be conducted [48]. Several studies used lung organoids as models for various lung diseases, such as cystic fibrosis, chronic obstructive pulmonary disease, and SARS-CoV-2 infection [49]. Selected examples of 3D lung organoids over the past 6 years are summarized in Table 2. More specifically, organoids using cystic fibrosis patient-specific induced pluripotent stem cells (iPSC)-derived lung epithelial cells can be used for drug screening [49]. Xu et al. confirmed that RIPK1, which is increased in COVID-19 patients who experience cytokine storm, was also activated in SARS-CoV-2 infection organoids and inhibited viral entry after treatment with RIPK-1 inhibitors [50]. Lung cancer organoids (LCOs) were implemented as a valid preclinical model with performance similar to a patient-derived xenograft model, and also established in a 3D culture system [51]. Surgically resected lung cancer tissues were dissociated into individual cells or cell clusters. These cells were then embedded in Matrigel and submerged in minimum basal media (Fig. 3A) [52]. Although research on lung organoids is increasing in various medical conditions, few studies have focused on lung organoids in TBI. Recently, robust lung organoids were shown to represent a tool for translational research to emulate parts of the lung-brain axis and interface with microbiota to confirm host-microbiome cross-talk [53]. Nevertheless, lung organoids are still difficult to approach for clinicians who treat patients with TBI. Accordingly, we would like to explain the concept and production of lung organoids. The main function of the human lung is gas exchange between air required by the human body and blood flowing through capillaries. Conventional 2D cell cultures are limited in recapitulating the structural characteristics of the lungs and their physiological activity because they lack a vascular network of immune and other cells, restricting their applications to disease modeling [54]. Moreover, the ethical responsibility of animal experimentation has led to increased alternative models and some do not have results relevant or translatable to humans [55]. Technology has advanced to offer various organoid cultures for endoderm-, mesoderm-, and ectoderm-derived organs. In particular, lung organoids using adult stem cells, ESCs, and iPSCs have been created to reproduce the alveolar structure, mucosal secretion, self-renewal, and self-organization abilities [56–59]. In generating lung organoids, various ECM materials, such as basement membrane extracts or collagen obtained from animal cells, alginate or agarose obtained from plant cells, and chemically synthesized hydrogels, can provide support for cell attachment and have been added to 3D cell cultures investigating the control of differentiation-related signaling pathways in lung cells *via* the air-liquid interface [60]. Van der Dose et al. developed a simple method to isolate alveolar type II (AEC2) cells from human lung tissues [61]. Large lung tissue samples from a lung cancer patient were dissociated to obtain single-cell suspensions. The single-cell suspension was obtained after straining through a 100 μm mesh. The selected AEC2s were resuspended in basement membrane extract (BME2) for subsequent seeding in a 48-well plate at 1 × 10^5^ viable cells/30 µL to induce droplet formation and incubated at 37 °C to solidify the droplets. After adding fresh alveolar organoid medium twice a week, alveolar organoids were generated (Fig. 3B). To verify the generation of alveolar organoids, surfactant proteins A and B, E-cadherin were used to stain the AEC2 organoids to verify the formation of lamellar body-like structures. In addition, LCOs are generated to simulate the physiological environment in the human body. Human primary tumor cells and human lung cancer cells were used in the culture of such organoids, and the application can be extended to basic studies on cancer origin and development and related fields of drug screening. Chung et al. developed LCO models using lung cancer tissues from the cryobiopsy samples of lung cancer patients [62]. LCOs were generated in solidified BME2 drops, and the expression patterns were found to be similar to those of the key markers of lung adenocarcinoma, including TTF-1, p40, and PD-L1, as shown in Fig. 3C. Moreover, the general morphology of LCOs showed pathological expression patterns that resembled major lung cancer tissue. Recently, lung organoids have been developed to analyze diseases caused by human respiratory infection with the influenza virus [63, 64]. Influenza virus refers to a negative-sense single-stranded RNA virus of the *Orthomyxoviridae* family, which can be applied in lung organoids for virus-to-host interactions and subsequent drug discovery. Rana et al. used alveolar lung organoids with high expression levels of hPSC-derived angiotensin-converting enzyme 2 and transmembrane protease serine 2 (TMPRSS2) in an alveolar model of SARS-CoV-2 infection [65]. The human pulmonary pathophysiology of SARS-CoV-2 infection was examined by differentiating iPSCs to definitive endoderms (DEs), then to anterior foregut endoderm/spheroids (AFEs) as lung progenitor cells, and finally to lung organoids. The AFEs showed an epithelial-like structure until 12 days of culture in the lung organoid medium, and mesenchymal populations were visually confirmed with the steady growth of ductal epithelial cells (Fig. 3D). The AFEs contained progenitor cells, such as SOX2^+^ cells, and the lung organoids exhibited a prominent epithelial structure up to 60 days of culture. The spike protein-mediated and TMPRSS2-dependent SARS-CoV-2 infection model with lung organoids was verified, and drugs that can prevent infection were tested by treating the cells with the TMPRSS2 inhibitor camostat, then quantifying luciferase activity. The results demonstrated the effect of camostat on preventing viral infections and that the infiltration of similar viruses was mediated by a spike protein in a TMPRSS2-dependent pathway. Looking at the research results to date, advanced lung organoids with an environment similar to the human body can maintain the fundamental physiological activity of lung cells, providing opportunities to understand the functional characteristics of human lungs and helping to overcome the limitations of conventional 2D cultures.

**Table 2:** Examples of established 3D organoids recapitulating key lung functions

Cell source	Transplanted time	Specific markers	Model	Methods for organoid generation	Result	Ref.
Human alveolar type II cells (HTII280+) or alveolar epithelial progenitor cells (HTII-280+, TM4SF1+) combined with human fetal lung fibroblast cell line	21 days	SFTPC + AQP5+	Alveolar organoids	Matrigel and Transwell	Lung progenitor cells and human AEPs (hAEPs) were directly isolated, expressing the conserved cell surface marker TM4SF1, and hAEPs acted as functional human alveolar epithelial progenitor cells in 3D organoids	[128]
Human lung cells	N/A	SFTPB+, SFTPC + KRT5 + AC-TUB + MUC5AC + CC10+	Lung organoids	Matrigel	Adult lung organoids (ALOs), primary airway cells, and hiPSC-derived alveolar type-II (AT2) pneumocytes were infected with SARS-CoV-2 to create in vitro lung models of COVID-19	[129]
Mouse fetal epithelial tips	6 days	SOX2 + SOX9 + FOXJ1 + KRT5 + SFTPC+, RAGE+	Alveolar organoids	Matrigel	Serum-free conditions allow for the growth and differentiation of mouse distal lung epithelial progenitors and chemical screening to investigate WNT signaling in epithelial differentiation	[130]
Rat fetal distal lung epithelial cells combined with CD31 + rat endothelial cells	15 days	RT2-70 + CC10 + EPCAM+	Lung organoids	Matrigel and ALI-culture	Alveolar and endothelial cells from fetal rat lung tissue were used to generate lung organoids in specific culture conditions, including a Matrigel gradient and air/liquid interface, and replicated key biological lung functions, including the presence of ion channels with highly selective cation (HSC) and non-selective cation (NSC) channel-like properties for lung maturation	[131]
Human pluripotent stem cells	27 days	EPCAM+, CPM+, NKX2.1 + AQP5+, T1α + SFTPC + vimentin+	Alveolar organoids	Matrigel	Generated alveolar organoids (AOs) derived from human pluripotent stem cells (hPSCs) for the anti-fibrotic mechanisms of potential drug efficacy evaluation	[132]
Human iPSCs	2, 6, and 16 weeks	NKX2.1+, SOX2 + SOX9 + SCGB1A1 + MUC5AC+	Lung organoids	Matrigel	Progenitor cells were then used to induce three-dimensional (3D) organoids derived from human pluripotent stem cells (hPSCs) with features resembling bud tips. The research revealed a high degree of similarity in the gene expression of lung epithelial markers among whole fetal lungs, fetal progenitor organoids, pulmonary-like organoids (PLOs), and bud tip organoids.	[133]
Human iPSCs	N/A	KRT5 + SCGB3A2 + MUC5AC + SP-C+, SP‐B+, HTII‐280+, AGER+	Lung organoids	Matrigel	Human embryonic stem cells (hESCs) and induced pluripotent stem cells (hiPSCs) were differentiated and formed 3D structures that emulated the development of lung organoids, confirming the production of surfactant and the presence of ciliated cells	[134]
Human embryonic stem cells	30 days	NKX2.1 + proSFTPB+, proSFTPC+	Embryonic cells alveolospheres	Matrigel	Generation of alveolar epithelial type 2 cells (AEC2s), the facultative progenitors of lung alveoli, from human PSCs. Purified PSC-derived SFTPC + cells formed monolayered epithelial “alveolospheres” in 3D cultures	[135]
Human embryonic lung distal tip cells	70–98 days	SOX2 + SOX9+, HMGA2+, ETV5+, HNF1B+	Lung epithelial tip organoids	Matrigel	Human and mouse tips were analogous and signaling pathways that are sufficient for the long-term self-renewal of human tips as differentiation-competent organoids were identified. Mouse and human differences, including markers that define progenitor states, were also identified	[136]

### Possible engineering platforms to recapitulate the brain-lung axis

**Fig. 4:**
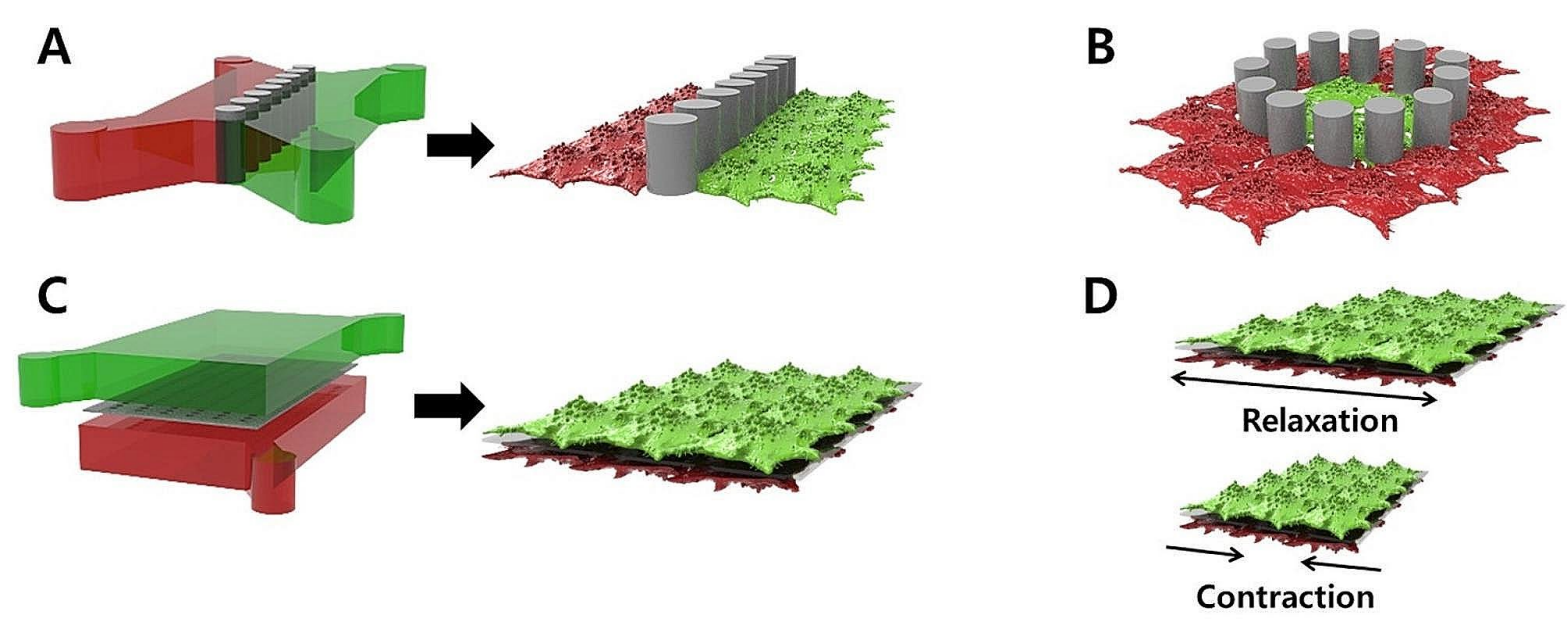
Microfluidic technique for the co-culture of different cells and organoids with in situ monitoring. **(A)** Schematic illustration of the parallel-type of microfluidic cell culture device with micro-pillar structures for investigating the interactions of different cell lines. **(B)** The parallel-type chip with structural modification for the development of more elaborate physiological environments. **(C)** Schematic representation of the vertical-type microfluidic cell culture device comprising the upper and lower cell culture chambers. **(D)** External stimulus-induced microfluidic cell culture device for the recapitulation of dynamic physiological environments

Researchers have used advanced biological systems to develop the interconnections needed to induce cellular communication between different cell lines, thereby generating potential in vitro multiorgan models. Recent advances of Organ-on-a-chip (OoC) have shown that can be developed biological model system to recapitulate the specific functions of various types of human tissues and organs [66]. This novel platform has enabled the realization of cell culture, as well as cell-to-cell and cell-to-ECM interactions, which mimic in vivo like physiological environments. In general, the conventional Transwell system has limitations in real-time monitoring and an accurate physiological environment, which were resolved by the development of a parallel-type OoC platform [67]. The use of human-derived cells has allowed various mechanisms in the human body to be mimicked and real-time monitoring and analyses to be performed. Wei Sun et al. co-cultured cancer cells and normal cells in a parallel-type microfluidic cell culture device for co-culturing different cells [68]. In this study, the OoC consisted of two chambers that allowed cancer cells and normal cells to be cultured in isolation while a microchannel connected the chambers midway (Fig. 4A). Breast cancer cells (MDA-MB-231) and human mammary epithelial cells were co-cultured at concentrations of 0.5 × 10^6^, 2 × 10^6^, and 6 × 10^6^ cells/mL, and different breast cancer models were developed. The viability and interaction of cancer cell and normal cell were monitored in real-time by identifying trends in the counts of migrating cells and the distance cancer cells migrated between the microchannels. The results showed that an increase in the density of cancer cells determined the probability of the incidence of metastatic cells and that the induction of normal cells had an effect on the migration distance of cancer cells. A blood-brain barrier (BBB)-on-chip is a biochip that can simulate the complex microenvironment at the BBB, which restricts the transport of materials between the brain and blood vessels for use in drug screening and studies on brain diseases. However, the conventional platform cannot recapitulate the blood flow dynamics in the actual vasculature, and more accurate in vitro models of brain disease are needed. Mir et al. developed a BBB model system in which uniform current density can be induced by controlling the position, dimension, and distance between the electrodes [69]. The center chamber consists the post structures in 100 μm intervals to mimic the BBB, and changes in the BBB were monitored in real-time using transepithelial/transendothelial electrical resistance (TEER) measurements. Hydrogel containing pericytes and astrocytes was injected into the central channel, and brain endothelial cells were injected at either end of the channel during the culture to form the BBB. The location, morphology, and survival capacity of each cell and the development of tight junctions (TJs) across endothelial cells were investigated by immunofluorescence analysis to demonstrate the ability of the in vitro brain model. BBB permeability was assessed by injecting the drug GNR-PEG-Ang2/D1 and identifying increases in the expression of VE-cadherin and Zo-1 of TJ proteins, demonstrating the potential of the BBB model system to promote drug screening for neurodegenerative diseases. Similarly, the slope-ALI microfluidic chip has physically separated compartments with an epidermal part for the culture of keratinocytes, two central ECM parts where the posts are, and a soma culture part where the neurons are located [70]. This created a skin barrier and collagen type-1 and collagen type-1/10% laminin mixture were injected into each ECM layer to ensure stable cell culture and increase neuronal activity. The OoC was also applied to simulate hyperglycemia-induced diabetic neuropathy caused by glucose at high concentrations, whereby ERK activation and cell proliferation in keratinocytes could be induced based on increased intracellular reactive oxygen species in neurons, as well as the reduced length and number of epidermal nerve fibers. Thus, the potential use of the chip as an in vitro model has been verified with the formation of thicker skin barriers and the spatial separation of cells, axons, and soma, which could not be achieved in conventional 2D co-culture systems. As shown in Fig. 4B, the modified parallel-type chip structure allows for the simulation of a more elaborate physiological environment through structural modifications. Kiani et al. developed a parallel-type chip with a trap-post structure to develop a dynamic neonatal BBB-on-chip that mimics the 3D microvasculature in the body [71]. The center of the OoC is a core compartment consisting of rat astrocytes, which is surrounded by a channel comprising cultured vascular cells. Depending on the length of the vascular channel, interactions with the compartment with cultured astrocytes can be induced through the interface with 3 μm pores arranged at set intervals. The expression of ZO-1 protein, with a role in the co-culture of rat brain endothelial cells and rat astrocytes, demonstrated that the biochip could be used as a BBB model, enabling more accurate vascular endothelial cell-to-brain cell interactions. Thus, the modified BBB-on-chip may allow a visual assessment of cell morphology and intercellular interactions *via* fluorescence staining for real-time monitoring. The biochip will also allow the reproduction of functions similar to the in vivo BBB by regulating shear stress related to blood flow to control physical stimuli or by simulating the characteristics of the BBB in brain disease by co-culturing with various cell lines. In cancer research, changes in the microenvironment can induce cancer progression and metastasis in numerous ways. However, previous in vitro studies on the tumor microenvironment and drug evaluation had limitations in creating the tumor microenvironment of cancer cells, their surrounding cells, and the ECM. Huh et al. developed a human breast cancer-on-a-chip that simulated the 3D tumor environment in ductal carcinoma in situ (DCIS) [72]. The biochip was constructed to be a vertical-type microfluidic cell culture device and a porous membrane coated with collagen type 1 was inserted between the microchannels of the upper and lower compartments (Fig. 4C). DCIS spheroids were cultured in the upper compartment, whereas breast cancer fibroblasts and stroma layers were formed in the lower compartment, generating the capillaries of actual breast stroma. Mammary epithelium growth medium and human mammary fibroblast culture media were introduced into the top and bottom channels with a constant volumetric flow for the co-culture. As a result, a tumor microenvironment allowing interactions between capillaries and the cultured multicellular DCIS spheroids of 200 μm was successfully realized, allowing *in-situ* monitoring and visualization of the respective biological reactions of early-stage breast cancer development. The progression of DCIS to malignancy and anticancer drug effects were evaluated by controlling the type and spatial distribution of the cells and the precise parameters of the signals in the microenvironment. Thus, the human breast cancer-on-chip was shown to allow the structural and functional simulation of tumor cells and other cells in breast ducts and soft tissue compartments, with outstanding reproducibility and spatial recreation of microenvironments similar to those in the human body, which will provide accurate physiological data in relevant research. Huh et al. developed a lung-on-a-chip with similar geometrical structures, which provided an opportunity to perform drug screening and investigate cellular responses to chemicals, particles, toxins, pathogens, and other environmental stimuli [73]. Compartmentalized Polydimethylsiloxane (PDMS) microchannels are present on a thin and flexible porous PDMS membrane for the formation of the alveolar-capillary interface. Human alveolar epithelial cells and microvascular endothelial cells were injected into the respective channels to develop the lung-on-a-chip, where they adhered to the surface of the ECM-coated membrane, and a single layer of cells expressing the binding proteins occludin and VE-cadherin, which connect endothelial cells, was created. Specifically, the device involves the mechanical extension of the PDMS membrane that forms the alveolar-capillary interface by applying a vacuum to the lateral chambers, as shown in Fig. 4D. This enables the reproduction of the physiological respiratory motion. Intrapleural pressure falls upon diaphragm contraction during inhalation in the body while the alveoli expand and the alveolar-capillary interface is physically stretched. A pulmonary nanotoxicology model was developed using the device presented in this study to investigate the cellular responses to lung inflammation and infection. Ultrafine silica nanoparticles were injected into the alveolar epithelium through the ALI; the neutrophils migrated on the surface of the epithelium and penetrated through the alveolar-capillary interface through the porous PDMS membrane. Ingber et al. presented a human orthotropic lung cancer-on-a-chip that was developed by introducing non-small-cell lung cancer (NSCLC) cells into the interior of a normal lung-on-a-chip [74]. The microfluidic chip with two vertical microchannels had human primary airway epithelial cells and H1975 NSCLC cells cultured in the upper part of a porous polyester-polyethylene terephthalate membrane and human lung microvascular endothelial cells cultured on the 4 walls of the lower channel to form a 3D hollow vascular lumen, generating an alveolar microenvironment. For the mechanical motion of respiration, the pressure applied to the lateral walls was adjusted to simulate the membrane contraction-relaxation movements, which could reproduce the specific behaviors of tumor cells in a normal lung microenvironment. Kim et al. developed a vertical-type BBB-on-a-chip for cell culture consisting of human brain microvascular endothelial cells (HBMECs) in the upper compartment and human brain vascular pericytes in the lower compartment [75]. The central channel in the lower compartment had a Matrigel/astrocyte mixture as the ECM. The expression levels of vimentin, an astrocyte expression factor, and lipocalin-2 (LCN2), an inflammatory mediator, were decreased compared to the conventional 2D culture model. This study demonstrated the use of a BBB-on-a-chip in examining the physiological behaviors of human astrocytes, and that the OoC can be applied to modeling astrogliosis associated with brain inflammation. Vertical-type OoC platforms can efficiently induce cell-cell interactions by generating an interface across different co-cultured cell lines to create physiological environments that resemble human organs. These platforms allow for the simulation of dynamic physiological environments in the human body through the use of external stimuli. Significant advancements in the diversity of OoC platforms are anticipated to recapitulate the physiological microenvironments to provide appropriate in vitro biological models.

### Controversial issues and future perspectives

As previously mentioned, pulmonary complications are the most common non-neurologic organ system failure in patients with TBI [76]. The reported ARDS incidence in isolated severe TBI is 7.5–25% [77–79]. Clinical outcomes, including hospital mortality, length of stay, the duration of mechanical ventilation, and tracheostomy placement, were worse in TBI patients with ARDS compared to those without ARDS [77]. In addition, lung injuries that develop in TBI patients not only increase healthcare utilization rates but also worsen the long-term neurological prognosis [80]. Lungs and the brain are an integrated ensemble that mutually influences each other as the lung-brain axis [81]. However, few studies have been conducted on the interaction between the lungs and brain, especially in patients with TBI, and generating a disease model that reflects this connection by approaching the organs simultaneously rather than individually is difficult. Although many studies have demonstrated that the brain and lungs can interact with each other through different pathways, including neuroanatomical [82–84], endocrine [85–87], immune [88–90], metabolites [91–93], microorganism [94, 95], and gas pathways [96, 97], an appropriate in vitro model system is still required. Exploring the interactions between the brain and lungs not only helps us study disease development from single- and multiorgan aspects but also provides important knowledge for treatment strategies (Table 3). We believe that brain-lung interactions based on organoids will help overcome the limitations of existing research.

**Table 3:** Overview of recently published brain-linked single- and multiorgan-on-a-chip platforms

Organ-on-a-chip	Cell types	Disease model	Functionalities and clinical tests	Ref.
Brain-on-a-chip	Primary hBMECs, primary human astrocytes, and pericytes, iPSC-derived neurons	BBB permeability	Permeability (FITC-dextran and ascorbate) TEER measurement	[137]
	Primary hBMECs, primary human astrocytes and pericytes, iPSC-derived neurons and astrocytes	NVU-under immune stimulation	ELISAs for cytokine release TEER measurement Metabolomic analysis	[138]
	Primary iBMECs, primary human astrocytes and pericytes, iPSC-derived neurons and astrocytes	Opioid transport	Drug permeability	[139]
	iBMECs and iPSC-derived EZ-spheres	Huntington`s disease	Permeability (fluorescence)	[140]
	iPSC-derived dopaminergic neurons	Parkinson`s disease	Proximity ligation assay	[141]
	iPSC-derived neurons	Alzheimer`s disease	Localization using fluorescence microscopy	[142]
	iPSC-derived neurons and astrocytes	Neurotoxin exposure	Cell viability Electrophysiology	[143]
Blood-brain barrier (BBB)-on-a-chip	iPSC-derived endothelial cells, human pericytes, and astrocytes	Drug transport in BBB	Permeability (FITC-dextran) 3D confocal imaging analysis	[144]
	iPSC-derived MBECs, human primary astrocytes, and pericytes	Drug transport in BBB	Permeability with ELISA and LC-MS/MS	[145]
	iBMECs, rat primary astrocytes	Drug transport in BBB	TEER measurement 3D confocal imaging analysis (TJ evaluation) Permeability (FITC-dextran and drug)	[146]
	Hypoxia-exposed iBMECs, primary human astrocytes, and pericytes	Drug transport in BBB	TEER measurement Permeability (FITC-dextran and drug) with ELISA and fluorescence measurement	[147]
	hiPSC-derived endothelial and astrocyte cells	Nitrosative stress, antioxidant prophylaxis	Real-time monitoring of barrier integrity Metabolomic network analysis	[148]
	iPSC-derived brain microvascular endothelial cells, neurons, primary human brain astrocytes, and pericytes	Huntington’s disease, MCT8 deficiency	Permeability and viability assay Immunostaining Gene ontology analysis	[140]
	Endothelial cells, pericytes, human neural stem cells, fungal	Fungal meningitis	Immunohistochemistry qPCR and RNA sequencing Permeability and viability assay Fungal BBB invasion assay	[149]
	iPSC-derived dopaminergic neurons, primary human astrocytes, primary human brain pericytes, primary human brain microglia, human brain microvascular endothelial cells	Parkinson’s disease	Mitochondrial membrane potentials assay Western blots and immunostaining assay Cathepsin D activity assay	[150]
Brain-linked multiorgan-on-a-chip	NTera-2/cl.D1 (NT2) cells (brain), HepaRG, and human hepatic stellate cells (liver)	Neurotoxicity	Lactate dehydrogenase (LDH) assay Real-time qPCR TUNEL assay and immunostaining Cell metabolic activity assay	[151]
	Ha-1800 cells (brain), human bronchial epithelial cells, A549 cells (lung), Fob1.1 cells (bone), L-02 cells (liver)	Invasion potential of lung cancer cells and associated fibroblasts in distant organs	Immunostaining (E-cadherin, N-cadherin, Snail1, Snail2, CD206, a-SMA, CEA) Hoechst and PI staining Fluorescence microscopy imaging analysis	[152]
	Human iPSC-derived neurons (brain), human iPSC-derived intestinal and stromal cells (intestine), human iPSC-derived hepatocytes (liver), human iPSC-derived renal cells (kidney)	Patient-specific multi-OoC model preparation	Immunostaining (ZO-1, 4 alpha, Cyt 8/18, vimentin, Ki-67, CdX2, TUBB3, PAX6, nestin, TBR1) qPCR, RNA sequencing Glucose and LDH assay	[153]
	Brain microvascular endothelial cells, brain vascular pericytes and astrocytes, microglia, neural cells (brain), human primary hepatocytes and Kupffer cells (liver), human iPSC-derived cardiomyocytes and cardiac fibroblasts (heart), A549 cells and human bronchial epithelial cells (lung), spermatogonial stem cells, Leydig cells, Sertoli cells, and peritubular cells (testis), cardiomyocytes, cardiac fibroblasts, cardiac endothelial cells (cardiac)	Parallel assessment of drug efficiency and toxicity in multiorgan models	Live and dead staining 3D confocal imaging analysis	[154]

*LC-MS/MS, liquid chromatography-tandem mass spectrometry; TEER, Transepithelial/transendothelial electrical resistance; ELISA, Enzyme-Linked Immunosorbent Assay

Research has recently increasingly reported on the relationship between various organs, not only brain-lung interactions [98]. From this point of view, the issue arises of how to incorporate other organs, in particular oral conditions, into the brain-lung organoid system. The oral and lung systems are directly interconnected through the airway. Oral and lung microbiota also exhibit similarities in healthy individuals [99]. Consequently, oral bacteria associated with periodontal disease might function as pulmonary pathogens through aspiration. Additionally, inflammatory cytokines, such as MMP-9, TNF-a, and IL-6, which are increased in periodontal disease, could potentially impact lung health through aspiration [100]. The oral-brain relationship parallels the oral-lung relationship. The oral microbiome can detrimentally affect the brain via the bloodstream or the trigeminal nerve [101, 102]. Another scenario where the oral environment can influence both the brain and the lungs is in the context of systemic low-grade inflammation. Elevated levels of C-reactive protein and pro-inflammatory cytokines like TNF-α and IL-6 induced by periodontal disease are linked to systemic disorders and could indirectly affect both the lungs and the brain [100, 103]. Consequently, when devising future brain-lung organoid models, it would be advantageous to incorporate factors related to oral conditions that could impact the organoids, thereby creating a more accurately simulated environment. However, it is important to note that current oral organoids are still in the conceptual stage and have only seen limited success in applications, such as salivary glands and tooth germs [104]. Thus, it becomes imperative to account for interactions between the oral-brain-lung systems or oral-lung interactions to replicate an environment where periodontal disease can occur. This necessitates a deeper comprehension of specialized oral structures, including bones, teeth, and periodontal tissues, where both hard and soft tissues are intricately connected biologically and physiologically [105]. Thus, when crafting a brain-lung organoid model, it appears more judicious to contemplate the inclusion of oral bacteria, their metabolome, or host immune-related factors that are influenced by the metabolome rather than attempting to incorporate entire oral organoids.

Many researchers have generated specific target organoids derived from ESCs or iPSCs. COs can be generated from neural ectoderm and lung organoids from embryonic endoderm [106]. The development of these organoids enables the modeling of certain diseases. Thus, organoid-based modeling can replace traditional animal preclinical tests and be used to study medicinal effects. In addition, 3D-organoid technologies can provide platforms to simulate interactions between host organs and infectious pathogens [106]. However, some limitations need to be overcome for actual clinical use. First, Matrigel, a non-human derived ECM, may cause immune responses due to viral or xenogenic contaminants. This is because it is produced by mouse Englebreth-Holm-Swarm sarcoma cells [106]. Moreover, there is variability in the biochemical properties of different batches, and even within the same batch, of Matrigel. This can complicate the reproducibility and quality control of organoid production. As a solution, novel hydrogels have been proposed to develop artificially synthesized ECM or nanofibrillar hydrogels for culturing organoids [107–109]. The second challenge is to produce organoids that accurately simulate diseases affecting multiple organs. Although several studies have demonstrated successful transplantation of various organoids into hosts, the current organoid culture systems have not yet sufficiently recapitulated the complexity of the human body. In particular, they have not effectively replicated the tissue-to-tissue interactions found in mature native tissues, especially those from different organs. Workman et al. suggested co-culture systems that can overcome the aforementioned limitations [110]. They generated human intestinal tissue containing a functional enteric nervous system by combining human iPSC-derived neural crest cells and human intestinal organoids. Moreover, recent studies have used organ-on-a-chip technologies to explore interactions between different types of organoids. Through multi-organ modeling in microfluidic culture conditions, new insights can be gained into the connections and communications between multiple organs [111]. For example, Jin et al. developed a microfluidic array culture system that incorporates liver, intestinal, and stomach organoids to create a multi-organ model for drug screening [112]. Similarly, Skardal et al. combined 3D organoids to investigate the feasibility of microengineered heart-lung-liver models [113]. Based on these studies, it is important to develop a co-culture platform to assess potential interactions between the brain and lungs, which is acrucial issue for patients with TBI. Thirdly, high reproducibility is essential for the clinical application of organoids. The characteristics of organoids depend on the source and passage of cells, leading to batch-to-batch variations in cellular composition, maturity, and architecture. Organoids derived from PSC tend to resemble fetal tissue rather than adult stem cell-derived organoids due to their limited lifespan for full development and maturation [106, 107]. In contrast, according to Clevers et al., adult stem cell-derived organoids exhibit lower structural complexity than PSC-derived organoids, but their architecture is closer to that of adult tissues [114]. To overcome these limitations, it is necessary to develop technologies to create organoid models that can be used for diagnosis and treatment in real clinical settings.

Despite the limitations mentioned above, innovative organoid technology has already proven useful in a wide range of applications, from scientific research to clinical approaches. Furthermore, organoid models show promise for exploring human diseases from a forward-looking perspective. Human organoids could properly reproduce aging in disease modeling. Korea is an aging society and ranks as the sixth longest life expectancy at birth [115]. In the future, this aging society will worsen, and the proportion of those aged ≥ 65 years is expected to increase from 18.4% in 2023 to 46.4% in 2070 [116]. In real clinical circumstances, the number of elderly patients with TBI is increasing faster than that of young patients with TBI due to injuries in traffic accident or sports activity. These findings suggest the need for studies on the brain-lung axis in TBI patients in consideration of age.

## Conclusion

Three-dimensional brain and lung organoids can be helpful in understanding interactions in the brain-lung axis simultaneously in TBI conditions, which is important in clinical practice but has not been studied much. Generating brain and lung organoids from various cell lines is crucial for overcoming the limitations of in vitro and in vivo studies, which for many years have used post-mortem tissues. Organoid technologies can bridge the gap between conventional 2D cell culture and animal models, enhancing therapeutic possibilities in their clinical applications. This approach would facilitate organ-on-a-chip platforms by recapitulating the physiological environments that closely mimic the human brain and respiratory system and be helpful in developing personalized models for patients with TBI. We believe that brain-lung axis modeling using organoids is promising as it allows for long-term cultivation and the maintenance of the phenotypical and functional characteristics of the human brain and lung. Harnessing the potential of brain-lung axis-mimicking organ-on-a-chips would accelerate the design of new treatments and the strategy of diagnostic methodologies in TBI patients with various other conditions.

## Data Availability

All data used in this study are public.

## Abbreviations

TBI: Traumatic brain injury
ALI: Acute lung injury
ARDS: Acute respiratory distress syndrome
IICP: Increased intracranial pressure
TNF: Tumor necrosis factor
CNS: Central nervous system
CO: Cerebral organoid
ESCs: Embryonic stem cells
PSCs: Ppluripotent stem cells
2D: Two-dimensional
NSCs: Neural stem cells
NPCs: Neural progenitor cells
ECM: Extracellular matrix
EB: Embryonic body
hPSCs: human PSCs
NE: Nneuroepithelial
iPSC: Induced pluripotent stem cells
LCOs: Lung cancer organoids
AEC2: Alveolar type II
BME2: Basement membrane extract
TMPRSS2: Transmembrane protease serine 2
AFEs: Anterior foregut endoderm/spheroids
OoC: Organ-on-a-chip
BBB: Blood-brain barrier
TEER: Transepithelial/transendothelial electrical resistance
TJs: Tight junctions
DCIS: Ductal carcinoma in situ
PDMS: Polydimethylsiloxane
NSCLC: Non-small-cell lung cancer
HBMECs: Human brain microvascular endothelial cells
LCN2: Lipocalin-2
